# Generation of Cancer Stem/Initiating Cells by Cell–Cell Fusion

**DOI:** 10.3390/ijms23094514

**Published:** 2022-04-19

**Authors:** Thomas Dittmar

**Affiliations:** Institute of Immunology, Center for Biomedical Education and Research (ZBAF), University of Witten/Herdecke, Stockumer Str. 10, 58448 Witten, Germany; thomas.dittmar@uni-wh.de; Tel.: +49-2302-926-165

**Keywords:** cell-cell fusion, cancer, cancer stem/initiating cells

## Abstract

CS/ICs have raised great expectations in cancer research and therapy, as eradication of this key cancer cell type is expected to lead to a complete cure. Unfortunately, the biology of CS/ICs is rather complex, since no common CS/IC marker has yet been identified. Certain surface markers or ALDH1 expression can be used for detection, but some studies indicated that cancer cells exhibit a certain plasticity, so CS/ICs can also arise from non-CS/ICs. Another problem is intratumoral heterogeneity, from which it can be inferred that different CS/IC subclones must be present in the tumor. Cell–cell fusion between cancer cells and normal cells, such as macrophages and stem cells, has been associated with the generation of tumor hybrids that can exhibit novel properties, such as an enhanced metastatic capacity and even CS/IC properties. Moreover, cell–cell fusion is a complex process in which parental chromosomes are mixed and randomly distributed among daughter cells, resulting in multiple, unique tumor hybrids. These, if they have CS/IC properties, may contribute to the heterogeneity of the CS/IC pool. In this review, we will discuss whether cell–cell fusion could also lead to the origin of different CS/ICs that may expand the overall CS/IC pool in a primary tumor.

## 1. Introduction

The concept of cell–cell fusion in cancer was already proposed more than a century ago by the German physician Otto Aichel [1]. He assumed (a) that the fusion of tumor cells and cancer infiltrating leukocytes could be an explanation for aneuploidy and (b) that the combination of different chromosomes and their qualitative differences could lead to a metastatic phenotype (for review, see [1,2,3,4,5,6,7]). Since then, the fusogenic capacity of cancer cells has been demonstrated in a variety of in vitro and in vivo studies. It was shown that cancer cells could either fuse with other cancer cells or normal cells, such as macrophages, fibroblasts, stromal cells, and stem cells, thereby generating tumor hybrid cells exhibiting an increased metastatogenic capacity, an enhanced resistance to chemo- and radiation therapy, and even properties of cancer stem/initiating cells (CS/ICs) (for review, see [3,4,5,6,7,8,9,10,11]). Moreover, tumor hybrids have been clearly identified in human cancer patients with a former bone marrow transplantation (BMT) history [12,13,14,15,16,17]. For instance, tumor hybrid cells with overlapping donor and recipient alleles were found in the primary tumor, lymph node metastases, and brain metastases of melanoma patients [13,14,15]. Likewise, Y-chromosome positive tumor hybrid cells were found in the primary cancer and the circulation of female pancreatic adenocarcinoma patients, which received a BMT from a male donor [17]. Moreover, the presence of circulating tumor hybrid cells as compared to normal circulating tumor cells was correlated to a statistically significantly increased risk of death in pancreatic cancer patients [17], suggesting that the cancer cells’ malignancy was dramatically enhanced by fusion. Briefly, these human cancer data support the theory that (a) cell–cell fusion events really occur in human cancers and that (b) cell–cell fusion could give rise to tumor hybrid cells exhibiting an altered phenotype.

CS/ICs represent a small population of cancer cells exhibiting stem-cell properties (for review, see [18,19,20,21,22,23,24]) and prospective CS/ICs have been identified in leukemia [25,26] and various solid tumors, including breast, prostate, pancreatic cancer, colorectal cancer, and glioblastoma [27,28,29,30,31,32]. In addition to their tumor initiating potential, CS/ICs may also induce metastases [33,34] and could be responsible for tumor relapse after therapy [35,36,37]. Hence, CS/ICs are of pivotal interest in cancer research and therapy, as their destruction would deprive the tumor of its source [35,38].

What sounds simple and promising on the one hand, is actually very complex. First, prospective CS/ICs are usually characterized by the expression of specific markers (for review, see [18,19,20,21,22,23,24]), but no common CS/IC marker has been identified so far. Moreover, a few studies suggest some plasticity of cancer cells, such that non-CS/ICs can become CS/ICs [39,40,41,42]. Second, the capacity of CS/ICs to induce tumor formation is studied in immunocompromised mice [43]. This is advantageous since defined mouse strains guarantee comparable and reliable results. In contrast, the murine tumor environment is barely comparable with the human tumor microenvironment. Thus, human CS/ICs will respond differently to murine cytokines, matrix components, or murine cells, which all have an impact on the cells’ tumorigenicity. Moreover, the tumorigenicity of CS/ICs is also influenced by co-implantation of, e.g., matrix components and stromal cells [19,44]. Third, the originally proposed CS/IC model cannot explain intra-tumoral heterogeneity, which is a hallmark of cancer [45]. Instead of a clone of more or less identical CS/ICs, which were derived from the initial CS/IC by symmetric division, it is currently assumed that the CS/IC pool within a tumor is more heterogeneous, suggesting that different CS/IC subclones co-exist and drive tumor progression [19,23].

This raises the question of how these different CS/IC subclones might have arisen. In addition to the conversion of non-CS/ICs to CS/ICs by various intrinsic and extrinsic cues acting simultaneously or independently [19], in this review, we will discuss whether cell–cell fusion could also lead to the origin of different CS/ICs that may expand the overall CS/IC pool in a primary tumor.

## 2. Some Brief Facts about CS/ICs

Even though prospective CS/ICs have been characterized by expression of specific markers, such as CD24, CD44, CD90, CD133, and ALDH1 (for review, see [18,19,20,21,22,23,24]), no common CS/IC marker has been identified so far and, for some CS/ICs, different markers have been proposed (Table 1). For instance, CD133 has been suggested as a marker for glioblastoma, pancreatic, and prostate CS/ICs [46,47,48], while putative breast CS/ICs have been designated as CD44^+^/CD24^−/low^ [27]. Interestingly, mouse mammary Brca1 tumors contained distinct CD44^+^/CD24^−^ and CD133^+^ cells with CS/IC characteristics [49], whereas three distinct triple negative breast CS/IC populations (both CD44^+^/CD24^−^ and CD44^+^/CD24^+^ in estrogen receptor α-negative breast tumors, and CD44^+^/CD49f^hi^/CD133/2^hi^) were tumorigenic in murine xenograft models [50]. Hermann and colleagues identified two distinct prospective CS/IC populations in pancreatic cancer. CD133^+^ CS/ICs were found in the center of the primary tumor, whereas CD133^+^ CXCR4^+^ CS/ICs were present in the invasive front of pancreatic cancer and determined the metastatic phenotype [48]. However, a contrastingly different CD44^+^CD24^+^ESA^+^ pancreatic CS/IC phenotype has been suggested by Li and colleagues [29]. Epithelial cell-adhesion molecule (EpCAM) and CD44 have been suggested as more robust colon CS/IC markers [31] than CD133 [51,52]. However, CD133^+^CD44^+^CD49^high^ colon cancer cells were highly tumorigenic in a study of Haraguchi and colleagues [53].

In addition to the inconsistency of prospective CS/ICs markers, various studies revealed that non-CS/ICs could be as tumorigenic as CS/ICs and that CS/IC-related markers were reversibly expressed in non-CS/ICs and CS/ICs [39,40,41,42]. For instance, CD271 has been suggested as a marker for melanoma CS/ICs [58], but both CD271^+^ and CD271^−^ melanoma cells were highly tumorigenic and metastatic in nonobese diabetic/severe combined immunodeficient (NOD/SCID) interleukin-2 receptor-gamma mice (NSG mice) [39]. CD133^+^ and CD133^−^ glioblastoma CS/ICs showed differential growth characteristics and molecular profiles, but both subtypes were similarly tumorigenic in nude mice [41]. Interestingly, Wang et al. showed that CD133^−^ glioma cells were tumorigenic in nude rats and could give rise to CD133^+^ cells [40], which have been suggested as glioblastoma CS/ICs [46]. While these findings point to a possible plasticity of cancer cells, these data again raise the reliability of certain markers in CS/IC research.

Side population cells (SP cells) represent another population of tumor initiating cancer cells [57,62,63,64,65]. They are characterized by expression of ATP binding cassette (ABC) membrane transporters and the efflux of fluorescent dyes, such as Hoechst blue and Hoechst red, which are usually used for detection and isolation [57,62,63,64,65]. SP cells have been identified in several tumor cell lines derived from, e.g., breast cancer [54], lung cancer [57], glioblastoma [56], and pancreatic adenocarcinoma [61], and human cancers, such as breast [54], melanoma [59], and osteosarcoma [60] (Table 1). While all these studies showed that SP cells were highly tumorigenic, it remains less clear whether SP cells are identical to the above-mentioned population of CS/ICs or represent a unique population of tumorigenic cancer cells. Studies on pancreatic cancer SP cells revealed that, at less than 1000 cells, tumor formation was initiated in nude mice, but that both non-SP cells and SP cells contained CD44^+^CD24^+^ and CD133^+^ cells [61]. As indicated above, CD44, CD24, and CD133 have been proposed as markers for prospective pancreatic CS/ICs [29,48]. Likewise, no correlation between breast cancer SP cells and the prospective breast CS/IC phenotype CD44^+^CD24^−/low^ was observed [54]. About 3.4% SP cells were present in the population of MCF-7/HER2 breast cancer cells and only 100 of these MCF-7/HER2 SP cells sufficiently initiated tumor formation in NOD/SCID mice. However, solely 7.4% of MCF-7/HER2 breast cancer cells were CD44^+^/CD24^−/low^ [54].

In 2008, two different studies suggested a link between epithelial-to-mesenchymal transition (EMT) and generation of normal stem cells and CS/ICs [55,66] (Table 1). The transition of sessile epithelial cells into motile mesenchymal cells is usually associated with cancer metastasis [67,68,69,70], but, of course, is also mandatory for developmental processes during embryogenesis and wound healing [71]. EMT is a reversible process and, hence, cells that have undergone EMT could revert to an epithelial state via mesenchymal-to-epithelial transition (MET) [70]. Several EMT-inducing triggers have been identified, such as transforming growth factor-β (TGF-β), WNT proteins, cytokines, growth factors, and hypoxia, which leads to the expression and functional activation of various master EMT regulators, specifically EMT-inducing transcription factors (EMT-TFs) and microRNAs [68,72]. In this context, two core EMT circuits/feedback loops have been identified, consisting of SNAIL-miR-34 and ZEB1-miR-200 [73], which are accompanied by additional EMT-TFs, including SLUG, TWIST, and YAP/TAZ, as well as post-translational modifications and splicing [71,72]. Interestingly, mathematical modeling of the two core EMT circuits revealed that cells could attain either an E, or an M or an E/M phenotype depending on their SNAIL expression levels [73]. It is this E/M phenotype, which has also been named hybrid E/M state [73,74,75] or quasi-mesenchymal [68], that likely possesses CS/ICs properties. For instance, single CD24^+^/CD44^+^ HMLER cells (human mammary epithelial cells immortalized and transformed with hTERT, SV40LT, and RAS oncogenes [76]) exhibited a hybrid E/M phenotype and possessed increased stem-like properties, such as an enhanced population of ALDH1+ cells and mammosphere formation capacity [74]. Likewise, tumorigenic cancer cells expressing both E- and M-specific markers have been found in xenografts isolated from breast [77] and ovarian tumors [78]. Data of Kroger and colleagues further indicated that the hybrid E/M phenotype in HMLER cells is likely controlled by SNAIL, whereas the M phenotype is driven by ZEB1 [79]. HMLER cells that were trapped in a hybrid E/M state (ZEB1 knock-out, SNAIL expression) produced much bigger tumors and displayed a 37-fold higher tumor-initiating frequency as compared to appropriate controls [79]. In this context, integrin-β4 (CD104) has been suggested as a marker for the hybrid E/M state in triple negative breast cancer. Indeed, human breast cancer cells with an intermediate level of integrin-β4 expression exhibited a hybrid E/M state and were more tumorigenic than breast cancer cells in the E-state or M-state, respectively [80].

Tumor initiation and self-renewal of prospective CS/ICs is usually studied in the so-called serial transplantation assay, which, however, is still imperfect, since human cancer cells are growing in a nonhuman environment, namely immunocompromised mice [43]. Hence, it remains unclear whether tumor formation was truly related to CS/ICs or to cancer cells, which adopted best to the foreign murine environment. Moreover, the tumor initiation capacity of prospective CS/ICs is strongly related to the used mouse model (detection of tumorigenic cells is several orders of magnitude higher in NSG mice than in NOD/SCID mice [81]) and co-implantation of matrix components and additional tumor propagating cells [82]. It is well known that the right composition of the tumor microenvironment and the CS/IC niche, including matrix components, stromal cells, immunocompetent cells, hypoxia, and cytokines, potentially contribute to stemness and an enhanced tumor initiation capacity [19,44]. In any case, CS/ICs were clearly identified in genetically engineered mouse models of brain, skin, and intestinal cancers [37,83,84], which are, to date, the strongest evidence that CS/ICs exist and initiate tumor growth.

In summary, the biology of CS/ICs is complex. Although various strategies and protocols have been developed over the past two decades to identify and characterize potential CS/ICs in human cancers, it is still not clear which method and which characterization is the best and most reliable. Moreover, the “classical CS/IC” model fails in intratumoral heterogeneity, which is a hallmark of cancer [45]. Based on the classical CS/IC model, there should be a population of CS/IC in the tumor that arose by self-renewal and should, therefore, be phenotypically similar. However, this is not the case. Instead, different subclones are likely found in the tumor, each of them most likely originating from one subclone-specific CS/IC [19,23].

However, how does this pool of subclone-specific CS/IC evolve? One possible mechanism could be the accumulation of genetic mutations in one CS/IC that could lead to the evolution of subclone-specific CS/ICs [23]. Likewise, it cannot be ruled out that subclone specific CS/ICs evolve independently of the original tumor-initiating CS/IC. As mentioned above, some cancer cells appear to exhibit some plasticity, so that non-CS/ICs can give rise to CS/ICs [39,40,41,42], which could be the result of a microenvironmental cue or a (epi-)genetic change [19]. The hybrid E/M state model assumes that cancer cells could acquire a CS/IC phenotype through EMT [68,73,74,75]. Since EMT is induced in several cancer cells it can be further assumed that a number of hybrid E/M state cancer cells will be generated exhibiting prospective CS/IC properties. Thus, the hybrid E/M state model suits better to intratumoral heterogeneity and the necessity of individual CS/IC subclones.

**Figure 1:**
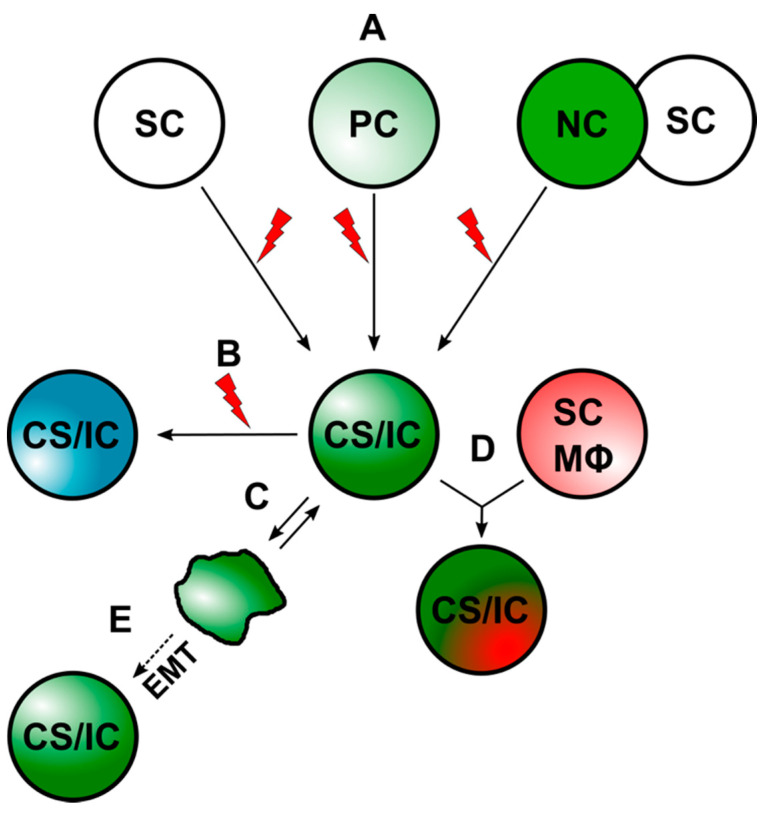
CS/ICs can arise through different mechanisms. (**A**) CS/ICs could originate from normal stem cells (SCs), progenitor cells (PCs), or through cell–cell fusion. The red lightning should indicate mutational events, which drive malignant transformation. (**B**) Mutational events could lead to a new CS/IC subclone. (**C**) Differentiated cancer cells could convert into CS/ICs due to an inherent plasticity. (**D**). CS/ICs could fuse with normal somatic cells (NC), such as stem cells (SC) and macrophages (Mϕ). (**E**) Induction of EMT in cancer cells might also give to a CS/IC phenotype.

Cell–cell fusion has been suggested as another possible mechanism by which prospective CS/ICs may arise [10,36,85,86,87,88,89,90]. Thereby, CS/ICs might originate from the fusion between stem cells and somatic cells [90] or tumor cells and normal cells, such as macrophages and stem cells [10,36,85,86,87,88,89,90]. A summary of the different ways that CS/ICs could arise is given in Figure 1.

Before we will review and discuss findings about cell–cell fusion and generation of tumor hybrid cells exhibiting prospective CS/IC properties, some brief facts about cell–cell fusion will be given.

## 3. Some Brief Facts about Cell–Cell Fusion

Cell–cell fusion is a hallmark for physiological processes, such as fertilization, placentation, myogenesis, osteoclastogenesis, and wound healing/tissue regeneration, and pathophysiological conditions, such as infection of host cells with enveloped viruses and cancer (for review, see [3,4,5,6,7,8,10,91,92,93,94,95,96]). While the merging of two (and more) cells appears simple, like two (and more) soap bubbles, cell–cell fusion is a highly complex, tightly regulated, energy-dependent, and still scarcely understood process [97]. This applies not only to the process of fusing two (and more) cells, but also to what happens to the hybrid cells afterwards.

**Figure 2:**
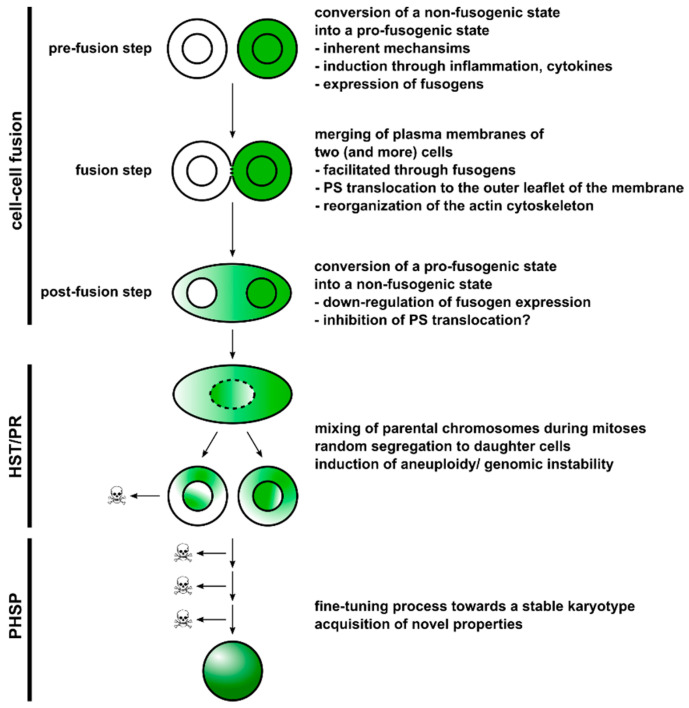
This scheme summarizes the different steps of cell–cell fusion and subsequent processes that might occur after hybridization. Cell–cell fusion is a complex and tightly regulated process, as cells must be converted to a pro-fusogenic state prior to fusion of plasma membranes and must then transition back to a non-fusogenic state. Heterokaryons could either remain in a bi- or multinucleated state or could undergo HST/PR, which is a mitosis-like process and characterized by merging of parental chromosomes and random segregation to daughter cells. HST/PR is associated with induction of aneuploidy and genomic instability, and the main reason why most tumor hybrids die or remain in a senescent state. Surviving and proliferating tumor hybrids fine tune their aneuploid/genomic instable karyotype during PHSP, which is also accompanied by cell death. Because of random segregation of parental chromosomes concomitant with further chromosomal aberrations during PHSP, tumor hybrids with novel properties could evolve.

Briefly, cell–cell fusion can be subdivided into three major steps, which are (a) the prefusion step, (b) the membrane fusion step, and (c) the post-fusion step [92,93,98] (Figure 2).

### 3.1. Prefusion Step

The prefusion step is characterized by a conversion of a non-fusogenic cellular state into a pro-fusogenic (Figure 2). It is associated with expression of the fusion machinery, such as chemokines/chemokine receptors, adhesion molecules, and fusogens, and alterations in the composition of the plasma membrane [91,92,93,95,98]. The induction of a pro-fusogenic phenotype can occur in two different ways. Certain cell–cell fusion processes occur during embryogenesis, such as placentation and myogenesis, suggesting an inherent, genetically/epigenetically-determined mechanism. Thus, the expression of certain cell–cell-fusion-relevant proteins is induced at specific differentiation time points in fusion-competent cells. For instance, human mononucleated cytotrophoblasts fuse together into syncytiotrophoblasts beginning at embryonic day 6 [99], which is mediated by the fusogens syncytin-1 and syncytin-2 [100,101]. Interestingly, syncytin-1 is continuously expressed in cytotrophoblasts until 37 weeks of gestation [102]. Expression of syncytin-1 and -2 is controlled by the embryonic transcription factor glial cell missing-1 (GCM1), which, in turn, is activated via cyclic adenosine monophosphate (cAMP)-dependent protein kinase A (PKA) signaling [103,104]. Conjointly, syncytin-2 expression could also be induced via a CRE/AP-1 motif [105]. Myoblast fusion is facilitated by the fusogens myomerger and myomerger, which are transiently expressed in embryonic development with peak expression levels during skeletal muscle development [106]. In mice, myomaker and myomerger share similar expression patterns with the two muscle-specific transcription factors MyoD and MyoG [107,108], which are targets of canonical Wnt/β-catenin signaling [109].

Wound healing/tissue regeneration represents another physiological process depending on cell–cell fusion [110,111,112,113]. However, in contrast to physiological fusion processes during embryogenesis, the merging of cells in wound healing/tissue regeneration must be induced at a certain time point. For instance, quiescent satellite cells are activated and undergo several state transitions upon muscle injury, including myomaker, myomerger, MyoD, and MyoG expression and cell–cell fusion [106]. Bone-marrow-derived cells (BMDCs), such as hematopoietic stem cells (HSCs), mesenchymal stem cells (MSCs), and cells of the myelomonocytic lineage, also exhibit fusogenic capacities and it was shown that they could merge with different cell types, such as hepatocytes [114,115,116,117], cardiomyocytes [115], neurons [115,118,119], and intestinal epithelial cells [120,121,122,123], thereby restoring tissue function. Hence, the fusogenecity of BMDCs is in a way different to physiological fusion events, such as fertilization, placentation, or osteoclastogenesis, as BMDCs could merge with several different cell types.

The ability to restore damaged tissues by cell–cell fusion requires the activation of the fusogenic machinery in cells, such as myoblasts and BMDCs and target cells. Inflammation is a well-known trigger for cell–cell fusion [118,120,124,125]. For instance, the frequency of binucleated heterokaryons derived from BMDCs and Purkinje neurons was 10–100-fold higher under chronic inflammatory conditions [118]. Wound healing/tissue regeneration/inflammation is orchestrated by discrete macrophage subtypes named classical activated/M1 macrophages and alternatively activated/M2a,b,c,d macrophages (for review, see [126,127]), which can be phenotypically distinguished from each other by their gene expression profile (surface markers and secretion of cytokines/chemokines) and those molecules that lead to their activation and differentiation [128,129]. Tumor necrosis factor-α (TNF-α), which is secreted by M1 macrophages, and interleukin-4 (IL-4) and interleukin-13 (IL-13), which induces the differentiation towards a M2a phenotype, have been linked to cell–cell fusion [88,124,130,131,132,133,134,135,136,137,138,139,140,141]. Studies on macrophages and breast epithelial cells and breast cancer cells revealed that TNF-α promoted cell–cell fusion via induction of MMP-9 expression [132,133]. In contrast, both syncytin-1 and its cognate receptor ASCT-2 were upregulated by TNF-α in oral squamous carcinoma cells and human umbilical vein endothelial cells [134], which would be in line with the crucial role of both proteins in cell–cell fusion. Interestingly, IL-4 induced macrophage fusion and giant cell formation was also dependent on MMP-9 expression [141]. Likewise, it is well-known that multinucleated giant cells derived from macrophages are a hallmark of chronic inflammation [124], suggesting that elevated IL-4 and IL-13 levels in (chronic) inflammatory conditions both promote macrophage differentiation towards an M2a phenotype and fusion. Increased syncytin-1 and annexin-V expression levels concomitant with an enhanced fusion frequency were observed in IL-4- and IL-13-treated PC3 prostate cancer cells [88]. Interestingly, elevated IL-4 and IL-13 serum levels were only found in the media of PC3 cells either co-cultured with primary prostate smooth muscle cells and primary murine myoblasts, but not in mono-cultured cells [88].

### 3.2. The Fusion Step

The membrane fusion step is self-explanatory and describes the merging of two distinct plasma membranes (Figure 2). To fuse, plasma membranes of two cells must be positioned first at a distance of not closer than ~10 nm [92]. The merging of the two plasma membranes, which is the ultimate fusion process, is facilitated by fusogens, representing a group of evolutionary optimized cell–cell-fusion-mediating molecules, such as syncytin-1, syncytin-2, myomaker, myomerger, EFF-1, AFF-1, Izumo1, CD9, and Juno [91,92,93,98]. Some of them, such as syncytin-1 and EFF-1, share similarities with retroviral fusogens [142,143], whereas myomaker is a seven-transmembrane protein [144] and Izumo1 a member of the immunoglobulin superfamily [145]. Thus, different cell–cell fusion strategies based on different proteins have evolved over the course of evolution, which may explain why this process is not yet well characterized. In any case, fusogens are a class of molecules that catalyze the overcoming of four energetic barriers and steric formations of three distinct lipid intermediates, which have been named the “hallmarks of cell-cell fusion” [92]. These are (a) the dehydration of contracting and bringing phospholipid heads to distances of close to 0 nm, (b) merging of the outer phospholipid monolayers (so-called hemifusion) via stalk and/or diaphragm intermediates, and c) opening and expansion of fusion pores from nanometer diameter to multiple microns [92].

However, just as fusogens are essential for cell–cell fusion, so are the corresponding receptors to which the fusogens bind. Lack of or altered expression levels of fusogens and/or their cognate receptor are correlated to impaired cell–cell fusion frequencies. For instance, knockout of either Izumo1 (sperm), or Juno or CD9 (both oocyte) is associated with infertility due to a defective cell–cell fusion ability [145,146,147]. Likewise, altered placental syncytin and its receptor ASCT2 expression levels were observed in placental development and pre-eclampsia [102]. Thereby, decreased placental expression of syncytin but not ASCT2 likely contributed to altered trophoblastic cell–cell fusion processes and disturbed placental function in pre-eclampsia [102]. Furthermore, myomaker-myomerger knockout embryos lacked multinucleated myofibers in almost all skeletal muscle regions, which further underlines the importance of both proteins in myoblast fusion [107]. In any case, the expression of a specific fusogen on one cell (e.g., donor cell) and the expression of the cognate receptor on another cell (e.g., recipient cell) ensures (a) a controlled fusion of these two cells and (b) prevents unwanted, uncontrolled cell–cell fusion events between different cellular entities.

In addition to fusogens, phosphatidyl serine (PS) has been suggested as a uniquely conserved signaling module in cell–cell fusion [95]. PS is commonly known as an eat-me signal for apoptotic cells and an attractor for blood clotting factors to initiate the blood coagulation cascade [148,149]. However, various studies demonstrated that an impaired translocation of PS from the inner leaflet to the outer leaflet of the plasma membrane was correlated with a diminished cell–cell fusion frequency of myoblasts [150], trophoblasts [151], macrophages [152], and the sperm [153]. The role of PS in cell–cell fusion is not yet clear. It is assumed that PS in the outer leaflet of the plasma membrane either directly promotes fusogenic restructuring of fusogens or triggers the assembly of the fusion machinery [95].

Rearrangement of the actin cytoskeleton and formation of actin-enriched podosome-like protrusions has been suggested as another mechanism mediating cell–cell fusion of myoblasts [154,155,156,157], trophoblasts [158], and pre-osteoclasts/macrophages [159,160]. It is assumed that actin-enriched podosome-like protrusions of fusion-competent cells invade and penetrate the plasma membrane of another cell, which then may induce fusion pore formation and the merging of the plasma membranes [161,162]. However, it remains unclear whether protrusions contribute causally to cell–cell fusion or are only involved in the process and the actual merging of cell membranes is mediated by fusogens. S2R+ cells remained non-fusogenic after expression of known components of Drosophila myoblast fusion, including cell adhesion molecules and actin cytoskeletal regulators, despite extensive cell adhesion and F-actin enrichment at cell–cell contact sites [163]. Cell–cell fusion was observed only after S2R+ cells were transduced with the *C. elegans* fusogenic protein Eff-1 [163].

### 3.3. Postfusion Step

Finally, the postfusion step is characterized by a conversion from a pro-fusogenic cellular state towards a non-fusogenic cellular state to prevent additional uncontrolled cell–cell fusion events [92,98,142] (Figure 2). In accordance with the not yet fully identified factors/conditions and intrinsic mechanisms that convert non-fusogenic cells into pro-fusogenic, the reverse process also remains enigmatic. Studies on muscle development revealed a transient expression of myomaker and myomerger in myoblasts [106], suggesting that one mechanism to terminate cell–cell fusion is the downregulation of the expression of fusogens and cognate receptors. The importance of PS in cell–cell fusion has been demonstrated in several studies and an impaired translocation of PS from the inner to the outer leaflet of the membrane was correlated to a reduced fusion frequency [95,150,151,152,153]. Hence, factors/conditions/pathways inhibiting this translocation mechanism might be another possibility for how cell–cell fusion could be terminated.

### 3.4. Heterokaryon-to-Synkaryon Transition/Ploidy Reduction and Post-Hybrid Selection Process

Cells can fuse in a homotypic and heterotypic manner. Homotypic means that two identical cell types, such as trophoblasts, myoblasts, and macrophages, fuse with each other, whereas different cell types, such as BMDCs and tissue cells or the sperm and the oocyte undergo heterotypic cell–cell fusion (for review, see [3,4,5,6,7,8,10,91,92,93,94,95,96]). While cell–cell fusion generally leads to the formation of multinucleated polyploid cells (so-called syncytia), such as syncytiotrophoblasts, myofibers, and osteoclasts, cell–cell fusion could also give rise to mono-nucleated cells, such as the fertilized oocyte, BMDC × tissue hybrid cells, and tumor hybrid cells (for review, see [3,4,5,6,7,8,10,91,92,93,94,95,96]). Interestingly, the generation of a mononuclear cell (synkaryon) from a bi- (or multinucleated) hybrid cell (so-called heterokaryon) through a process, which has been termed heterokaryon-to-synkaryon transition (HST)/ploidy reduction (PR) (HST/PR) [90,164,165], is not attributed to a fusion-like merging of the discrete parental nuclei. Instead, it is characterized by an active cell cycle, resolution of the nuclear membranes, mixing of the parental chromosomes, and their segregation to daughter cells [165,166] (Figure 2).

Because bi- and multinucleated polyploid cells possess two and more copies of centrosomes (one from each parental cell), the outcome of each division depends on centrosome location, spindle reorganization and attachment to chromosomes, and completion/failure of cytokinesis [165]. A bipolar mitosis of a bi-nucleated cell could give rise to two daughter cells with a diploid karyotype, but could also result in two daughter cells with an aneuploid karyotype due to, e.g., mitotic checkpoint defects, cohesion defects, or merotelic attachments [6,166,167,168,169,170]. Likewise, multiple spindle poles could be formed, which would favor multipolar divisions and the production of highly aneuploid daughter cells [6,166,167,168,169,170]. Moreover, multipolar divisions could also be associated with merotelic attachments resulting in lagging chromosomes, micronuclei formation, and chromothripsis [171,172]. In summary, HST/PR could lead to the induction of aneuploidy and an overall enhanced genomic instability in tumor hybrid cells [168,169,173].

Whether the first daughter cells derived from tumor hybrid cells will survive is not clear, as this depends on the set of chromosomes they received during division and if aneuploidy is tolerated. Daughter cells need a minimum number of chromosomes ensuring that all physiological cellular processes keeping a cell alive are running properly, such as metabolism, DNA replication, protein expression, endocytosis and exocytosis, or vesicle transport. Likewise, aneuploidy must be tolerated, since chromosome mis-segregation and induction of aneuploidy is usually accompanied by irreversible cell cycle arrest, impaired proliferation, and/or cell death [168]. Thus, daughter cells derived from tumor hybrids must express proteins and activate pathways that facilitate survival and tolerance to stresses induced by aneuploidy [168]. However, even if the first daughter cells survive, there is no guarantee that their progenies will do so in subsequent divisions. An aneuploid/genomic instable parental karyotype always gives rise to aneuploid/genomic instable daughter karyotypes and so on. This process, which has been termed “post-hybrid selection process (PHSP)” [6], runs as long as the cells have acquired a more or less stable karyotype (Figure 2). It should be noted that PHSP occurs uniquely in each tumor hybrid cell and its progenies, ultimately resulting in different tumor hybrids with different chromosome numbers and aberrations. Even if the majority of tumor hybrid cells do not survive this process, the surviving tumor hybrid cells can contribute significantly to the heterogeneity of the tumor tissue. Mathematical modeling showed that, despite a rather low frequency of spontaneous somatic cell–cell fusion events of about 6.6 × 10^−5^ in vivo and the impact of spatial constraints, fusion-mediated recombination can have a profound impact on somatic evolution through the accelerated diversification of tumor cell populations and the generation of rare mutational variants capable of exploring larger swathes of adaptive landscapes [174]. Given that each surviving tumor hybrid cell could be the founder clone of an evolving landscape, it might be speculated that each of them exhibits prospective CS/IC properties.

## 4. Cell–Cell Fusion and Generation of CS/ICs

In addition to an enhanced metastatic phenotype and increased resistance to chemotherapy and radiotherapy, some studies suggest that prospective CS/ICs or CS/IC-like cells may also result from cell–cell fusion [85,86,87,88,89,175,176,177,178,179,180,181,182,183,184,185,186]. Thereby, CS/ICs or CS/IC-like cells might either originate from non-transformed cells by cell–cell fusion or from the hybridization of pre-existing tumor cells with other cells. The latter mechanism could lead to the increase in the existing pool of CS/IC subclones in the primary tumor (Figure 1).

### 4.1. Cell–Cell Fusion and Generation of Primary-Tumor-Initiating CS/ICs

A few studies have been published demonstrating that the fusion of two nontumorigenic cells can give rise to tumorigenic hybrid cells [183,184,185]. While these findings suit well to the assumption that cell–cell fusion could lead to an altered phenotype of hybrids, it must be critically noted that only the overall tumorigenicity of the hybrid cells was investigated in each study and no serial transplantation assay was conducted. For instance, up to 5 × 10^6^ hybrid cells were implanted in nude mice in a study by Duelli et al. [183], whereas 1 × 10^5^ cells were implanted in NSG mice in a work of Delespaul and colleagues [185]. As indicated above, the tumorigenic capacity of prospective CS/ICs greatly varies between different mouse strains. On the contrary, the finding that tumor formation was observed indicates that the injected cells contained a certain population of CS/ICs. Moreover, explanted tumor cells derived from tumorigenic hybrids were also able to reinduce tumor formation in a second mouse [183], likely suggesting that the tumorigenic hybrid cells were capable of self-renewing, which is another predictor for CS/ICs.

### 4.2. In Vitro Studies Supporting That Cell–Cell Fusion Can Give Rise to CS/ICs

Studies demonstrating that prospective CS/ICs or CS/IC-like cells may arise by cell–cell fusion can be subdivided into in vitro [86,88,175,181] and in vivo studies [17,85,89,176,177,178,179,180,182,186]. In in vitro studies, prospective CS/ICs or CS/IC-like cells were characterized by appropriate in vitro stem cell assays, such as sphere and colony formation assay, and expression of CS/IC-related markers, such as surface proteins, stemness transcription factors, and ALDH1 [86,88,175,181]. In the work of Gauck et al., five tumor hybrid clones (designated M13HS-X; X = clone number) derived from human HS578T-Hyg breast cancer cells and human M13SV1-EGFP-Neo breast epithelial cells exhibiting stem cell properties were investigated [181]. Even though all M13HS hybrids were derived from the same parental cells, each M13HS hybrid clone exhibited unique characteristics. For instance, the frequency of the ALDH1-positive cells was highest in M13HS-2 hybrids (~13.7%), whereas the mean mammosphere formation capacity was rather low and comparable to HS578T-Hyg breast cancer cells [181]. In contrast, most mammospheres were derived from M13HS-1 and -4 tumor hybrids, which contained only moderate numbers of ALDH1-positive cells (M13HS-1: ~6.6% and M13HS-4: ~4.2%). Interestingly, even though no ALDH1-positive population was found in M13HS-7 hybrids, cells were able to form mammospheres [181]. Co-cultivation of fully transformed RST IMR90 human fetal lung fibroblasts and partially transformed E6E7 IMR90 human fetal lung fibroblasts also resulted in unique tumor hybrids [86]. In accordance with Gauck et al., each tumor hybrid clone exhibited a unique ALDH1-positive population and sphere formation capacity, which were not correlated to each other [86]. About 20% ALDH1-positive cells and a CS/IC frequency in spheres of 1/ 37.5 were determined for clone H1. In contrast, a comparable ALDH1 frequency was observed for clone H4, but the CS/IC frequency in spheres was only about 1/1281 [86]. Uygur and colleagues hypothesized that prostate cancer cells could acquire CS/IC properties and an enhanced drug resistance through fusion with muscle cells [88]. Co-cultivation of PC3 prostate cancer cells and primary human myoblasts yielded in higher IL-4 and IL-13 serum levels, which induced syncytin-1, annexin V, and CD133 expression in PC3 cancer cells that was further correlated with an enhanced fusion frequency [88]. Interestingly, the fraction of CD133^+^ cells was positively correlated with the presence of fused cells [88]. In addition to the increased expression of the prospective prostate CS/IC marker CD133 [47], tumor hybrids also exhibited a decreased E-cadherin expression, but increased protein levels of AKT, MMP9, and vimentin, suggesting that hybrids may have undergone EMT [88]. This would be in line with the suggested link between EMT and a CS/IC phenotype [55,66]. However, no animal studies have been performed so far and, thus, the tumorigenicity of PC3 × human primary myoblast hybrids remains unclear. Briefly, in vitro studies are suitable for initial characterization of tumor hybrids but are not sufficient to conclude for a prospective CS/IC phenotype, which must be tested in animals.

### 4.3. In Vivo Studies Supporting That Cell–Cell Fusion Can Give Rise to CS/ICs

Within the past decade, several articles have been published demonstrating that tumor hybrids could be more tumorigenic and metastatogenic than the parental cancer cells [17,85,89,176,177,178,179,180,182,186]. While this is in line with the hypothesis that cell–cell fusion could give rise to prospective CS/ICs, it has to be critically noted that, in most studies, only the overall tumorigenicity of the tumor hybrids was investigated (Table 2).

For instance, polyethylene glycol (PEG)-generated tumor hybrids derived from SV40 immortalized nontumorigenic GES-1 gastric epithelial cell and cord matrix-derived MSCs initiated tumor formation in nude mice [85]. Likewise, PEG-derived MCF-7 × U937D_2_ and MDA-MB-231 × U937D_2_ tumor hybrids were both tumorigenic and metastatogenic in NOD/SCID mice [186]. Of note, nontumorigenic MCF-7 breast cancer cells became tumorigenic and metastatic upon fusion with M2-macrophage-like U937D2 cells, gained a CD44^+^CD24^−/low^ phenotype, and overexpressed epithelial–mesenchymal-transition-associated genes [186].

MDA-hyb1 and -hyb2 tumor hybrids were derived from spontaneous fusion events between human MDA-MB-231 breast cancer cells and human MSCs from cord blood explants [179]. Thereby, MDA-hyb1 and -hyb2 tumor hybrids generated larger tumors in NOD/SCID mice and were more metastatic than parental MDA-MB-231 breast cancer cells [179]. For instance, metastases were already detectable after 18 days in spleen, heart, kidney, lung, and liver of NOD/SCID mice, whereas no metastatic spreading was observed for the parental breast cancer cell line in this time frame [179]. Interestingly, expression analysis of MDA-byb1 and -hyb2 tumor hybrids revealed appropriate upregulation of EMT-associated genes, such as SLUG, collagen, fibronectin, N-cadherin, MMP3, and MMP9 [179], possibly indicating that tumor hybrids might have acquired a mixed E/M state. A serial transplantation assay, which is still the gold standard in CS/IC research [43], has only been performed in the studies of Wang et al. and Xu and colleagues [177,182]. In both studies, the fusion of weakly malignant cancer cells with stem cells gave rise to highly tumorigenic tumor hybrids [177,182]. For instance, injection of 5 × 10^4^ HepG2 × embryonic stem cell hybrids facilitated tumor formation in nude mice, whereas the injection of 5 × 10^4^ HepG2 hepatic carcinoma cells did not induce this effect [177]. Likewise, qPCR analysis suggested higher expression levels of CD44, CD133, ALDH1A1, and EpCAM in tumor hybrids than in HepG2 parental cancer cells [177]. Indeed, these findings likely indicate the impact of cell–cell fusion on the generation of tumor hybrids with a putative CS/IC phenotype. However, Wang and colleagues used human embryonic stem cells for their research and cell–cell fusion was artificially induced by laser treatment [177], which cannot be transferred to the in vivo situation. In contrast, spontaneously formed MSC × lung cancer hybrids were investigated in the work of Xu and colleagues [182]. In accordance with the data of Wang et al. and in line with the cell–cell fusion hypothesis, tumor hybrids were more tumorigenic than the parental lung cancer cell lines A549, H460, and SK-MES-1 [182]. As few as 1 × 10^3^ tumor hybrids induced tumor formation in nude mice, whereas a 100-fold higher cell number was required for parental lung cancer cell lines to initiate tumor growth [182]. Moreover, tumor hybrids lost their epithelial morphology and assumed a fibroblast-like appearance, which was accompanied by downregulation of E-cadherin and pan-cytokeratin, and upregulation of vimentin, α-smooth muscle actin, and fibronectin [182]. In addition to an EMT phenotype, qPCR data further indicated an increased expression of stemness markers, such as OCT4, Nanog, BMI1, Notch1, ALDH1, and Sox2, which are responsible for regulating and maintaining the stem cell phenotype [182]. Even though no secondary tumor formation studies have been performed here (which is usually conducted for proving in vivo self-renewal capacity of prospective CS/ICs [43]), these data strongly support the hypothesis that CS/ICs or CS/IC-like cells can originate by cell–cell fusion.

In addition to an enhanced overall tumorigenicity, the metastatic capacity of tumor hybrids was also investigated in some studies [17,178,179]. Given that only CS/ICs harbor tumor initiation potential, it can be concluded that this pivotal cancer cell population should also initiate metastatic lesions. PEG-derived tumor hybrids generated from Lewis lung cancer (LLC) cells and murine MSCs possessed a comparable tumor formation capacity as LLC cells, but were more metastatic [178]. Likewise, PEG-derived HepG2 × MSC hybrids initiated more metastases in the liver and lung of nude mice than parental HepG2 liver carcinoma cells [180]. As indicated above, MDA-hyb1 and -hyb2 tumor hybrids exhibited an enhanced metastatic behavior and formed secondary lesions in various organs within 18 days [179].

A general weakness of most studies is that tumor hybrids were generated ex vivo, which is attributed to fact that tumor hybrids can be much more easily isolated, propagated, and characterized in in vitro studies. Nonetheless, detection of tumor hybrids in vivo is feasible, but the proof and the experimental setting is more complex (Table 3).

Melzer and colleagues co-injected differently labeled MDA-MB-231 breast cancer cells (mCherry positive) and MSCs (GFP positive) in NOD/SCID mice and found double fluorescing tumor hybrids in the primary tumor [187]. Albeit the number of tumor hybrids was less than 0.5% of the tumor population, indicating that cell–cell fusion is rather a rare event [187], these results indicated that cell–cell fusion really occurs in vivo. However, tumor hybrids were not further characterized and, hence, it remains unclear whether they exhibited CS/IC properties. Using a GFP BMT prostate cancer model, Luo et al. investigated the role of BMDCs in prostate cancer progression [176]. Interestingly, BMDCs were recruited into mouse prostate tissues during tumorigenesis and, in some mice, a dual expression of GFP and prostate-cancer-specific marker was observed in prostate cancer cells [176]. Moreover, the volume of tumors harboring tumor hybrids was significantly higher than that of the control group at most time points [176], suggesting that double-positive prostate tumor hybrids were originated by cell–cell fusion and drove prostate cancer progression. However, BMDC × prostate cancer tumor hybrids were not isolated from primary tumors and, hence, it remains unclear whether they also possessed CS/IC characteristics.

Park and colleagues identified a highly tumorigenic CD34^+^ liver CS/IC population isolated from PLC/PRF/5 hepatoma cells [188]. Only 100 CD34^+^ liver CS/ICs induced liver cancer formation in NSG mice, whereas 1 × 10^6^ CD34^−^ liver cancer cells were needed [188]. Since CD34 is not expressed by liver cells, the question arose as to where this surface marker came from. CD34 is a well-known human HSC marker [189] and it is also well known that BDMCs could regenerate liver by cell–cell fusion [116,190], suggesting that CD34^+^ liver CS/ICs may have formed by cell–cell fusion. However, CD34 is not expressed by murine HSCs, but rather by multipotent and oligopotent progenitors [191], indicating that one of these progenitor populations was the fusion partner. In any case, the finding that a 1000-fold lower cell number sufficiently induced tumor formation in mice strongly supports the assumption that CD34^+^ liver cancer cells exhibited CS/IC properties.

CD34^+^ liver CS/ICs co-expressed liver-specific markers, such as OV6 and alpha-fetoprotein, and myelomonocytic markers, including CD14, CD31, CD45, and CD68 [89]. Likewise, xenografts derived from CD34^+^ liver CS/ICs expressed liver-specific enzymes, such as cytochromes and UDP-glucuronosyltransferases; secreted albumin; and also expressed myelomonocytic-specific cytokines, such as interleukin-1β, interleukin-6, and TNF-α; and chemokines, including interleukin-8, CCL2, and CCL5 [89]. Interestingly, CD34^+^ liver CS/ICs also exhibited phagocytic activity [89], which might be considered as another indicator that these cells may have originated by cell–cell fusion. In the work of Gast et al., three different experimental strategies were applied to monitor in vivo cell–cell fusion in murine cancers [17]. RFP/GFP tumor hybrids were clearly identified in tumors derived from H2B-RFP-expressing murine melanoma B16F10 cells that were intradermally injected in actin-GFP mice [17], indicating that cells have fused in vivo. Moreover, several macrophage antigens, such as CD45, CD11b, Csf1R, and F4/80, were expressed by RFP/GFP tumor hybrids [17], suggesting that tumor cells have preferentially fused with macrophages. Likewise, in vivo cell–cell fusion of melanoma cells and macrophages was further visualized by a Cre-LoxP-based strategy. Therefore, H2B-RFP/Cre melanoma cells were intradermally injected into R26R-stop-YFP transgenic mice and resulted in the generation of YFP-expressing tumor hybrids [17]. Conjointly, injection of fl-dsRed-fl-eGFP-expressing B16F10 melanoma cells into LysM-Cre mice also gave rise to eGFP-expressing tumor hybrids, which were originated by cell–cell fusion and subsequent Cre-mediated recombination [17]. Furthermore, 300 FACS-isolated in-vivo-derived hybrid cells were injected intradermally into secondary recipient mice and resulted in tumor growth, demonstrating that tumor hybrids retained tumorigenicity [17]. Secondary tumor formation is part of the serial transplantation assay to demonstrate the self-renewal capacity of prospective CS/ICs in vivo [43]. Thus, the finding that tumor hybrids retained tumorigenicity strongly suggests a prospective CS/IC phenotype. Moreover, in-vivo-derived tumor hybrids displayed different rates of tumor growth, indicating that tumor hybrids were phenotypically heterogeneous among themselves [17]. This is consistent with the observation that the fusion of tumor cells and normal cells invariably gives rise to tumor hybrid cells with different phenotypes. Moreover, it can be concluded that tumor hybrids indeed contribute to the increase in the intratumoral pool of CS/IC subclones and that each CS/IC subclone has a specific contribution to tumor progression. The finding that cell–cell-fusion-derived tumor hybrids possessed prospective CS/IC characteristics was further supported by metastasis formation studies. Interestingly, preferentially RFP^+^/GFP^+^/CD45^+^, but not RFP^+^/GFP^−^/CD45^−^ tumor cells were found in the circulation of animals [17], suggesting that cell–cell-fusion-derived tumor hybrids exhibited an enhanced metastatic capacity. This is consistent with findings that significantly more lung metastases were derived from tumor hybrids than from parental nonfused melanoma cells [17]. Given that metastatic cancer cells must also exhibit CS/IC properties to initiate secondary lesions, the data of Gast et al. strongly indicate that cell–cell fusion can give rise to tumorigenic and metastatic tumor hybrids with a prospective CS/IC phenotype.

## 5. Conclusions

An increasing body of evidence indicates that cell–cell fusion events could occur in cancer, which could give rise to tumor hybrids exhibiting novel properties, such as an enhanced drug resistance, an increased metastatic capacity, and even prospective CS/IC properties [12,13,15,16,17,36,87,88,89,121,136,174,176,177,178,179,180,181,182,183,185,186,187,192,193,194,195,196,197,198,199,200,201,202,203,204,205,206,207,208,209,210,211,212,213,214,215,216,217,218,219,220,221,222,223,224,225,226,227,228,229,230,231,232,233,234,235,236,237,238,239]. However, the biology of CS/ICs is complex, since no classical CS/IC markers have been identified so far. Instead, a panel of different proteins/markers, such as surface markers, ALDH1, ABC membrane transporters, and EMT-related proteins, have been characterized that can be used for classification of prospective CS/IC (for review, see [18,19,20,21,22,23,24]). Likewise, cancer cells with a non-CS/IC phenotype can differentiate into CS/ICs due to inherent plasticity [39,40,41,42]. Moreover, the tumor initiation capacity of prospective CS/ICs is commonly studied in immunocompromised mice, which is a suitable, but still imperfect model. It is well known that the overall tumorigenicity of prospective CS/ICs strongly depends on the used mouse strain and whether cells were co-implanted with, e.g., matrix components and/or stromal cells [81,82]. Likewise, only those injected cancer (stem/initiating) cells which have adopted best to the murine environment will initiate tumor formation.

**Figure 3:**
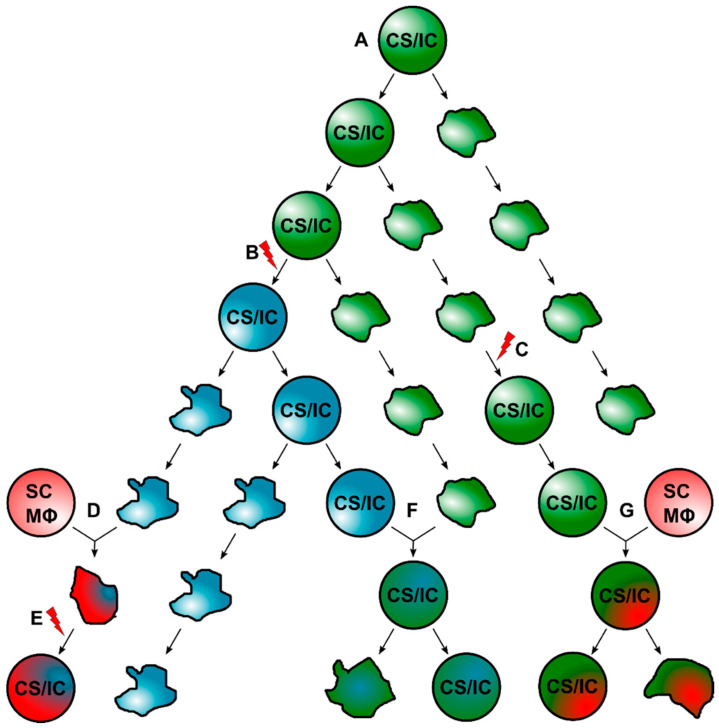
Promotion of intratumoral heterogeneity through cell–cell fusion and generation of CS/ICs. (**A**) Primary tumor formation is initiated by the initial CS/IC, which could self-renew and can give rise to differentiated proliferating cancer cells. (**B**) Due to mutational events, a new subclone CS/IC could originate from the initial CS/IC. (**C**) A non-CS/IC could convert into a CS/IC due to intrinsic/extrinsic processes, such as EMT. (**D**) Cancer cells could fuse with normal cells, such as stem cells or macrophages, thereby giving rise to tumor hybrid cells, which then (**E**) might gain a CS/IC phenotype due to intrinsic/extrinsic processes. (**F**) Intratumoral fusion of an existing subclone CS/IC and a tumor cell results in the formation of a new subclone CS/IC. (**G**) A subclone CS/IC fuses with a normal cell, such as a stem cell or a macrophage, which also results in the origin of a new subclone CS/IC. The red lightning should indicate mutational events.

Within the past years, it became evident that the classical CS/IC model is not compatible with intratumoral heterogeneity. Hence, the classical CS/IC model was extended and is now more dynamic, assuming that primary tumors harbor different CS/IC subclones [19,23]. The evolution of these diverse CS/IC subclones is less clear. CS/ICs could acquire additional genomic aberrations, which could lead to a new CS/IC subtype (Figure 1 and Figure 3). Likewise, normal cancer cells could convert into CS/ICs due to intrinsic and extrinsic alterations [19] and/or induction of EMT [68]. Finally, CS/IC subclones may also originate by cell–cell fusion [10,36,85,86,87,88,89,90] (Figure 1 and Figure 3).

If we assume that cell–cell fusion can give rise to tumor hybrids with CS/IC properties, this implies that a certain proportion of tumor hybrids must survive. As mentioned, HST/PR and PHSP, in particular, represent processes in which the majority of cells die or become senescent. However, even if the proportion of surviving tumor hybrids is small, they may contribute to tumor progression and intratumoral heterogeneity. This has been demonstrated in mathematical modeling, which revealed that, in comparison to mutations, cell–cell fusion can have a profound impact on somatic evolution through the accelerated diversification of tumor cell populations and the generation of rare mutational variants capable of exploring larger swathes of adaptive landscapes [174]. Given that each surviving tumor hybrid cell could be the founder cell for a new intratumoral landscape, it might be concluded that this particular tumor hybrid cell should possess CS/IC properties (Figure 3). As indicated above, tumor hybrids could be more tumorigenic and metastatogenic than the parental cancer cell line, which would be in line with the CS/IC model. Likewise, evolving tumor hybrids differ (markedly) among each other [86,179,181,185,210,240], which would be further in line with the finding that cell–cell fusion is a potent driver for intratumoral heterogeneity and the assumption that each evolving tumor hybrid cell could exhibit CS/IC properties.

Whether tumor hybrids fulfill all CS/IC criteria [43] is not clear, since this has not yet been investigated. The tumorigenicity of tumor hybrids in different cell numbers has been investigated in only three studies to date [177,182,188], but without investigating the self-renewal capacity of tumor hybrids, which, however, is another criteria that prospective CS/ICs have to fulfill [43]. In contrast, secondary tumor formation capacity of tumor hybrids was demonstrated by Gast et al., but no tumorigenicity studies using different dilutions of tumor hybrids has been investigated [17]. Nonetheless, 300 tumor hybrids sufficiently induced tumor formation, indicating that tumor hybrids exhibited a markedly enhanced tumor initiation capacity, which is a hallmark of CS/ICs [43]. Conjointly, tumor hybrids were phenotypically different, which supports the hypothesis that different evolving landscapes are initiated by CS/IC subclones.

If we assume that an increased tumorigenicity and (metastatogenic capacity) of tumor hybrids could be interpreted as possessing potential CS/IC properties, it could be inferred that cell–cell fusion represents a corresponding mechanism of how CS/ICs can arise and likely expand the intratumoral pool of CS/IC subclones. However, further studies would be needed to fully clarify this, and the potential CS/IC properties of individual tumor hybrids should be investigated in serial transplantation assays. Although this assay is not optimal, no other assays exist to date that can be used to investigate both the tumor initiation capacity and self-renewal ability of tumor hybrids. In vitro assays are helpful for initial characterization of tumor hybrids for potential CS/IC properties. However, due to the lack of defined CS/IC markers, the actual tumorigenicity of tumor hybrids cannot be deduced from these data. This can only be conducted in vivo.

In conclusion, cell–cell fusion is a mechanism from which tumor hybrids with new properties can arise, including CS/IC characteristics. These can increase the intratumoral pool of CS/IC subclones, promoting intratumoral heterogeneity and tumor progression. Thus, the process of cell–cell fusion would potentially represent a target for future cancer strategies. However, this requires knowledge of the molecular mechanism by which hybridization of tumor cells and normal cells can be prevented.

## Figures and Tables

**Table 1:** Prospective CS/IC marker.

Cancer Type	CS/IC Marker	Reference
Breast cancer	CD44^+^/CD24^−/low^	[27,49,50]
	CD44^+^/CD24^+^	[50]
	CD44^+^/CD49f^hi^/CD133/2^hi^	[50]
	CD133^+^	[49]
	ALDH1	[28]
	SP cells	[54]
	EMT	[55]
Colon Cancer	CD44^+^EpCam^+^	[31]
	CD133^+^	[51,52]
	CD133^+^CD44^+^CD49^high^	[53]
Glioblastoma	CD133^+^	[46]
	CD133^+^/CD133^−^	[40,41]
	SP cells	[56]
Lung Cancer	SP cells	[57]
Malignant Melanoma	CD271^+^	[58]
	CD271^+^/CD271^−^	[39]
	SP cells	[59]
Osteosarcoma	SP cells	[60]
Pancreatic Cancer	CD44^+^CD24^+^ESA^+^	[29]
	CD133	[48]
	SP cells	[61]

**Table 2:** In vitro cell–cell-fusion-derived tumor hybrids with prospective CS/IC characteristics.

Cancer Type	Fusion Partners	Tumorigenic/ Metastatogenic	CS/IC Marker	STA *	References
Breast cancer	MDA-MB-231 × MSCs	Yes/Yes	n.d. **	No	[179]
	MDA-MB-231 × U937D2 MCF-7 × U937D2	Yes/Yes	CD44^+^/CD24^−/low^	No	[186]
Gastric Cancer	GES-1 × MSCs	Yes/n.d.	n.d.	No	[85]
Liver Cancer	HepG2 × ESCs	Yes/No	CD44^+^, CD133^+^, ALDH1A1^+^, EpCAM^+^	Yes	[177]
	HepG2 × MSCs	No/Yes	n.d.	No	[180]
Lung Cancer	A549/H460/SK-MES × MSCs	Yes/No	EMT	Yes	[182]
	LLC × mMSCs	Yes/No	n.d.	No	[178]
Pancreatic Cancer	B16F10 melanoma × BMDCs	n.d./Yes	n.d.	No	[17]
Prostate Cancer	Prostate cancer cells × BMDCs	Yes/Yes	n.d.	No	[176]

* STA: serial transplantation assay; ** not determined.

**Table 3:** In vivo cell–cell-fusion-derived tumor hybrids with prospective CS/IC characteristics.

Cancer Type	Fusion Partners	Tumorigenic/ Metastatogenic	CS/IC Marker	STA *	References
Breast cancer	MDA-MB-231 × MSCs	n.d. **/n.d.	n.d.	No	[187]
Liver Cancer	PLC/PRF/5 hepatoma cells × BMDCs	Yes/n.d.	CD34^+^	No	[89,188]
Pancreatic Cancer	B16F10 melanoma × BMDCs	n.d./Yes	n.d.	No	[17]
Prostate Cancer	Prostate cancer cells × BMDCs	Yes/Yes	n.d.	No	[176]

* STA: serial transplantation assay; ** not determined.

## Data Availability

Not applicable.

## Abbreviations

ABC	ATP binding cassette
BMDCs	bone marrow-derived cells
BMT	bone marrow transplantation
CS/ICs	cancer stem/initiating cells
cAMP	cyclic adenosine monophosphate
EMT	epithelial-to-mesenchymal transition
EMT-TFs	EMT-inducing transcription factors
EpCAM	epithelial cell-adhesion molecule
GCM1	glial cell-missing-1
HSCs	hematopoietic stem cells
HST	heterokaryon-to-synkaryon transition
IL-4	interleukin-4
IL-13	interleukin-13
MET	mesenchymal-to-epithelial transition
Mϕ	macrophage
MSCs	mesenchymal stem cells
NC	normal cell
NOD/SCID	non-obese diabetic/severe combined immunodeficient
NSG mice	NOD/SCID Interleukin-2 receptor-gamma mice
PHSP	post-hybrid selection process
PKA	protein kinase A
PEG	polyethylene glycol
PC	progenitor cell
PR	ploidy reduction
PS	phosphatidyl serine
SC	stem cell
SP cells	side population cells
STA	serial transplantation assay
TGF-β	transforming growth factor-β
TNF-α	tumor necrosis factor-α
